# Smartphone Apps for Measuring Human Health and Climate Change Co-Benefits: A Comparison and Quality Rating of Available Apps

**DOI:** 10.2196/mhealth.5931

**Published:** 2016-12-19

**Authors:** Rachel K Sullivan, Samantha Marsh, Jakob Halvarsson, Michelle Holdsworth, Wilma Waterlander, Maartje P Poelman, Jennifer Ann Salmond, Hayley Christian, Lenny SC Koh, Janet E Cade, John C Spence, Alistair Woodward, Ralph Maddison

**Affiliations:** ^1^ National Institute for Health Innovation School of Population Health The University of Auckland Auckland New Zealand; ^2^ Faculty of Medicine Linköping University Linköping Sweden; ^3^ School of Health and Related Research Section of Public Health University of Sheffield Sheffield United Kingdom; ^4^ Department of Human Geography and Spatial Planning Utrecht University Utrecht Netherlands; ^5^ School of Environment Faculty of Science The University of Auckland Auckland New Zealand; ^6^ School of Population Health and Telethon Kids Institute The University of Western Australia Perth Australia; ^7^ Centre for Energy, Environment and Sustainability Management School University of Sheffield Sheffield United Kingdom; ^8^ Nutritional Epidemiology Group Schools of Food Science and Nutrition University of Leeds Leeds United Kingdom; ^9^ Faculty of Physical Education and Recreation University of Alberta Edmonton, AB Canada; ^10^ Epidemiology and Biostatistics School of Population Health The University of Auckland Auckland New Zealand; ^11^ Institute for Physical Activity and Nutrition Deakin University Victoria Australia

**Keywords:** climate change, noncommunicable diseases, smartphone apps, travel, diet, greenhouse gas emissions, carbon footprint, individual, behavior change

## Abstract

**Background:**

Climate change and the burden of noncommunicable diseases are major global challenges. Opportunities exist to investigate health and climate change co-benefits through a shift from motorized to active transport (walking and cycling) and a shift in dietary patterns away from a globalized diet to reduced consumption of meat and energy dense foods. Given the ubiquitous use and proliferation of smartphone apps, an opportunity exists to use this technology to capture individual travel and dietary behavior and the associated impact on the environment and health.

**Objective:**

The objective of the study is to identify, describe the features, and rate the quality of existing smartphone apps which capture personal travel and dietary behavior and simultaneously estimate the carbon cost and potential health consequences of these actions.

**Methods:**

The Google Play and Apple App Stores were searched between October 19 and November 6, 2015, and a secondary Google search using the apps filter was conducted between August 8 and September 18, 2016. Eligible apps were required to estimate the carbon cost of personal behaviors with the potential to include features to maximize health outcomes. The quality of included apps was assessed by 2 researchers using the Mobile Application Rating Scale (MARS).

**Results:**

Out of 7213 results, 40 apps were identified and rated. Multiple travel-related apps were identified, however no apps solely focused on the carbon impact or health consequences of dietary behavior. None of the rated apps provided sufficient information on the health consequences of travel and dietary behavior. Some apps included features to maximize participant engagement and encourage behavior change towards reduced greenhouse gas emissions. Most apps were rated as acceptable quality as determined by the MARS; 1 was of poor quality and 10 apps were of good quality. Interrater reliability of the 2 evaluators was excellent (ICC=0.94, 95% CI 0.87-0.97).

**Conclusions:**

Existing apps capturing travel and dietary behavior and the associated health and environmental impact are of mixed quality. Most apps do not include all desirable features or provide sufficient health information. Further research is needed to determine the potential of smartphone apps to evoke behavior change resulting in climate change and health co-benefits.

## Introduction

Reducing the impact of climate change on our planet and reducing the burden of noncommunicable diseases (NCDs) are major global challenges [1,2]. A strong link exists between climate change and public health, which can be demonstrated by measuring greenhouse gas (GHG) emissions. For example, increased use of motorized transport leads to increased GHG emissions, thereby contributing to climate change as well as reducing physical activity levels, which has been linked to the development of many NCDs [3-7]. Furthermore, our current food production system is one of the most important contributors to global GHG emissions, where certain dietary changes (eg, eating less meat and fewer calories) can benefit the environment and public health, especially for adult populations in high-income countries [3,8-10]. The unique connection between these 2 issues presents an opportunity to make changes which have co-benefits to health and the environment. Transport and diet are 2 areas in which behavioral change may offer the greatest potential for reducing the impact of both climate change and NCDs [5,7].

It is estimated that transport accounts for approximately 22% of the world’s energy-related carbon emissions due to increased reliance on motorized transport within both developed and developing countries [11-14]. While motorized transport may be time-efficient in the modern world, it contributes to the problem of physical inactivity and sedentary behavior, which have been identified as major behavioral risk factors contributing to many NCDs and their determinants, causing approximately 3.2 million deaths per year [3,4]. The World Health Organization predicts that 7.7% of the total mortality risk within high income countries is attributed to physical inactivity alone, with a further 8.4% associated with being overweight or obese [15]. However, the prevalence of physical inactivity and the associated burden of chronic disease could be lowered with small changes to individual travel behavior, such as reduced vehicle use and increased active travel (ie, walking or cycling) within urban areas [5,7,12-14,16-18]. Such changes also reduce the impact of climate change by lowering GHG emissions.

Agriculture accounts for approximately 31% of global GHG emissions with most emissions coming from livestock production, including methane from ruminant digestion, nitric oxide from fertilizer use, and carbon dioxide from deforestation/felled vegetation and fossil fuel use [6,19]. Livestock production is expected to rise substantially over the coming years to meet rapidly growing demands driven by population growth, economic growth, and urbanization within low and middle income countries, thereby exacerbating the effects of climate change [20]. In addition to this, globalization has seen increases in diets characterized by high energy (calories), saturated fat, free sugars, and salt, commonly associated with obesity and the development of many NCDs such as type 2 diabetes, ischemic heart disease, and some cancers [4,10,21]. Not only are these diets detrimental to health, they tend to focus on a narrow range of food crops, which increases the vulnerability of food supply to diseases, pests, and weather extremes that may arise with climate change, thereby also threatening food security [2]. Therefore, a universal shift away from a globalized diet toward reduced consumption of animal products and energy dense foods not only has the potential to reduce the burden of NCDs [4,8,9], it has also been predicted to significantly reduce GHG emissions due to a reduction in livestock production and associated emissions as well as increased land availability suitable for growing alternative food or the regrowth of native vegetation [22].

To date, studies of ways to reduce GHG emissions within transport and agricultural sectors have mainly focused on high-level modulations of potential policy changes [5,6,12,14,16] or technological and managerial approaches such as improving productivity, restoring soil carbon, optimizing nutrient use and fertilizers, improving livestock diets, and better management of waste [10,19,20]. However, little research has been done to examine the ways in which personal behavior affects both GHG emissions and the incidence of NCDs [17,19,23]. Although at the individual level the net effect of changing lifestyles (eg, changing 2 daily commutes per week from driving to cycling) might be relatively small, the impact at the population level could be substantial [14,16]. For example, studies show that if 10% of Canadians who are currently inactive or sedentary swap to active commuting by walking instead of driving and sit less, it could result in cost savings to the health care system of CaD $2.6 billion (US $2.0 billion) by 2040 and a cumulative CaD $7.5 billion (US $5.7 billion) boost to the Canadian gross domestic product [24]. Modeled data from New Zealand also showed that shifting 5% of vehicle kilometers to cycling would reduce vehicle travel by approximately 223 million kilometers each year and reduce transport-related GHG emissions by 0.4%, along with reduced NCD-related mortality [7].

A clear need exists for cost effective, minimal intervention strategies to promote and motivate individual behavior change. These strategies should focus on simple clear steps such as “leave the car at home today” [7,16] and “eat a vegetarian meal” [21,25]. Individual knowledge of small simple steps that shrink personal carbon footprints and reduce disease risk may increase peoples’ awareness and willingness to engage with large-scale problems such as climate change and obesogenic environments, which otherwise appear impossibly daunting and remote [26]. It is therefore important to address individual level behavior in addition to top-down approaches assessing the impact of policy changes and managerial or technological interventions. Such a bottom-up approach may prove to be more successful in addressing both health and environmental issues.

Traditional Web-based carbon calculators or surveys, requiring memory recall and manual input of personal behaviors, often result in nonuse, low adherence, and therefore inaccurate or incomplete data [27,28]. However, given the ubiquitous use and availability of smartphones, potential exists to use smartphone apps to capture personal travel and dietary behaviors, while simultaneously estimating the carbon cost and health benefits of these actions through new matrices and integrative carbon-health computation methodology.

Smartphones have advantages for collecting travel data compared with traditional surveys or Global Positioning System (GPS) loggers because as they are always taken with the user and have multiple inbuilt sensors such as GPS and accelerometers allowing automatic and continuous data collection, thereby creating a vast source of data (“big data”) for immediate data analytics, feedback, and decision making. For example, these data can be used to determine the frequency and duration of motorized transport and active travel and thereby estimate personal carbon emissions. For diet, technologies exist to assess food consumption and purchasing habits [29-31], which could be adapted to estimate the associated carbon cost.

To identify relevant smartphone apps which estimate the carbon cost and health impact of personal travel and dietary behavior and possibly promote changes in these behaviors, we reviewed free and paid smartphone apps to describe their features and rate the quality of these apps against a valid quality rating tool.

## Methods

### Search Strategy

A list of smartphone apps for both iOS and Android operating systems was compiled between October 19, 2015, and September 18, 2016. The Apple App Store (version 12.1.3) and the Google Play Store (version 6.9.21, using the apps filter) were searched between October 19 and November 6, 2015. A secondary Google search using the apps filter was conducted between August 8 and September 18, 2016, to identify any apps that may not have been available on the New Zealand app stores. This search was carried out using Mozilla Firefox (version 45.1.0) ensuring the researcher was logged out of their personal account and searching the web (rather than limiting the search to New Zealand) for both free and paid apps. Within the app stores, independent search phrases were used to identify relevant apps (Table 1). The method of searching used in Google to identify apps is shown in Table 2.

**Table 1:** Search terms used and results generated within Google Play store and Apple App Store to identify diet and transport-related apps (each search statement represents a separate search).

Search term	Results App Store	Results Google Play	Downloaded
Carbon footprint	71	233	16
Carbon calculator	39	16	2
Carbon AND food	3	124	1
Carbon AND diet	0	44	0
Environment AND food	0	250	0
Environment AND diet	1	250	1
GHG^a^ AND food	0	9	0
GHG AND diet	0	11	0
GHG calculator	0	9	0
CO_2_^b^ AND food	0	42	2
CO_2_ AND diet	0	11	0
CO_2_ calculator	36	114	1
CO_2_ emissions AND diet	0	4	0
Emissions food	1	148	1
Emissions diet	0	27	0
Greenhouse gas AND food	0	33	0
Greenhouse gas AND diet	0	65	0
CO_2_ tracker	12	109	0
Emissions calculator	17	250	1
Greenhouse gas calculator	1	25	0
Travel carbon emissions	8	86	7
Transport carbon emissions	0	132	2
Travel carbon footprint	7	62	2
Transport carbon footprint	0	74	1
Greenhouse gas emissions AND travel	0	53	0
Greenhouse gas emissions AND transport	0	66	1
GHG travel	0	21	0
GHG transport	0	28	0
CO_2_ emissions travel	13	72	4
CO_2_ emissions transport	1	112	1
Travel CO_2_	100	74	4
Transport CO_2_	100	121	0
Carbon AND travel	3	126	1
Carbon AND transport	0	138	0
Active transport CO_2_	0	5	0
Sustainable transport	4	202	5
Sustainable transport CO_2_	0	12	0
Sustainable travel	27	250	0
Sustainable mobility	3	118	0
Transport type	100	249	0
Transport type AND carbon emissions	0	25	0
Transport type AND carbon footprint	0	14	0
Transport diary	3	128	1
Active transport	2	250	2
Active transport AND CO_2_	0	6	0
Green transport	43	20	0
Commute CO_2_	1	17	0
Commute carbon emissions	1	19	0

^a^GHG: greenhouse gas

^b^CO_2_: carbon dioxide

**Table 2:** Search terms used for secondary Google search using the apps filter.

Search #	Search terms	Results	Downloaded
1	Carbon emissions AND health	60	5
	Carbon emissions AND travel	93	3
	Carbon emissions AND food	51	0
	Carbon emissions AND diet	5	0
	Search c & d	16	0
	Search a & b & e	15	0
2	Carbon emissions AND food AND travel	33	1
3	Health AND carbon emissions AND diet AND activity	19	1
4	Carbon footprint AND health	62	0
	Carbon footprint AND diet	18	0
	Carbon footprint AND food	70	2
	Carbon footprint AND travel	87	1
	Carbon footprint AND transport	63	1
	Search d & e	59	0
	Search a & f	18	0
	Search a & c	43	0
	Search g & h	5	0
5	CO_2_^a^AND health	96	2
	CO_2_ AND travel	78	1
	CO_2_ AND food	102	0
	CO_2_ AND diet	43	0
	Search a & b & d	11	0
6	Greenhouse gas emissions AND travel	32	1
	Greenhouse gas emissions AND transport	57	0
	Greenhouse gas emissions AND food	16	0
	Greenhouse gas emissions AND diet	6	0
	Greenhouse gas emissions AND health	24	0
	Search a & b & c & d & e	5	0

^a^CO_2_: carbon dioxide

### Inclusion Criteria

Potentially relevant apps were identified after careful consideration of titles and descriptions. Apps included in this review were in English, captured individual level travel or dietary behavior, and estimated the associated carbon cost, potentially including features to maximize health and reduce the burden of NCDs.

Due to the limitations (such as nonuse, low adherence, incompleteness, and inaccuracy) associated with manual input of personal travel behavior using carbon calculators or surveys, we searched for travel-based apps that captured personal travel data automatically and reported the associated carbon impact. However, auto-geolocation used in these apps has several limitations such as significant battery power consumption and privacy concerns, reducing its acceptability to users [28]. The majority of travel-based apps currently available are not fully automated but require users to manually initiate and terminate trip tracking or require completely manual data entry. Therefore, to be comprehensive in our search, travel-based apps were included if they recorded personal travel behavior automatically or with a manual start/stop and reported the associated carbon impact or required manual input to compare the carbon cost of different travel modes to help users make more sustainable travel choices.

Inclusion criteria for dietary apps were less specific. As dietary behavior cannot be easily recorded automatically, we searched for any apps that recorded some aspect of an individual’s dietary behavior in the estimation of personal GHG emissions, with potential to include information and/or tips to reduce diet-related emissions and improve health.

### Exclusion Criteria

Simple carbon calculators for transport emissions (ie, those requiring input of distance, type of fuel, and fuel consumption or engine efficiency) were excluded due to the limitations associated with manual data capture. Apps tracking travel location but not transport methods (ie, GPS tracking apps) were also excluded as they were unable to determine personal GHG emissions. Carpooling or taxi rideshare apps and health/fitness/”lifelog” style apps were also excluded if they did not include any reference to personal carbon emissions.

Out of the possible 7213 apps returned in searches, 28 potentially relevant transport-based apps and 34 dietary apps were downloaded. Following download, apps were reviewed to identify relevance for inclusion in the final sample. Of the 40 relevant apps, 11 transport-based apps and 4 dietary-based apps were offered on both iPhone and Android operating systems. In these cases, both versions were downloaded to check for consistency across both operating systems.

### App Quality

Apps were rated for quality using the Mobile Application Rating Scale (MARS). This scale was developed at the Queensland University of Technology, Australia, following a comprehensive review of Web- and app-related quality rating scores [32]. The MARS includes 4 domains, engagement, functionality, aesthetics, and information, and provides an overall mean score of the 4 domains. A separate subjective score assesses the user’s overall satisfaction, and an app specific score assesses the app’s ability to produce changes in the user’s awareness, knowledge, understanding, attitudes, and behavior.

All apps identified during the app store searches were reviewed and rated by 2 researchers (RS and JH) on either an iPhone 5S or an iPad for iOS and a Samsung Galaxy S5 Mini or Samsung Galaxy Note II for Android. Apps identified during the secondary Google search were rated by 1 researcher (RS). Apps that were consistent across both operating systems were only rated on iOS; however, if differences in layout or content were detected both Android and iOS versions were rated.

Before rating, online training videos [33] were reviewed to ensure correct use of the MARS scale, and the modified scales were tested using 3 carbon calculators not included in this review. Travel apps were typically used for at least 2 days to record travel activity, and dietary apps were used for a minimum of 15 minutes prior to rating.

### Analysis

All analysis was undertaken using SPSS Statistics version 21 (IBM Corp). Descriptive scores were calculated from the MARS scale. Independent sample, equal variance *t* tests identified the significance of any differences between travel and dietary apps or free and paid apps. Interrater reliability for the scores of the 2 researchers was calculated using the intraclass correlation coefficient (ICC). A 2-way mixed, absolute agreement, average measures model estimated the reliability of average measures between the 2 researchers.

## Results

### Search

**Figure 1:**
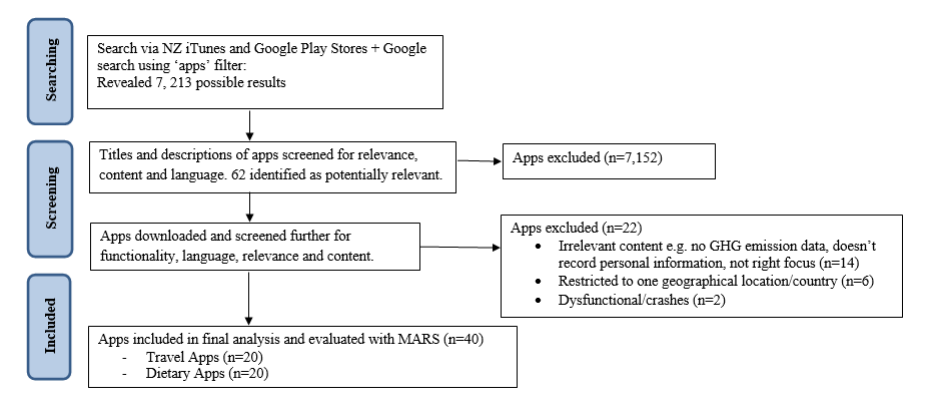
Flow chart of the systematic process for determining apps for inclusion in the final sample.

The procedure for identifying relevant apps for inclusion is shown in Figure 1. Of the apps downloaded, 8 travel-based apps and 14 dietary apps were excluded from further analysis. Out of 22 apps, 14 apps (64%) were irrelevant for our purposes (ie, did not include dietary information, did not capture individual level behavior/purely educational, or did not include carbon emissions data), 6 apps (27%) were restricted to certain geographical locations or countries, and 2 apps (9%) did not work (ie, crashing or having problems contacting the activation server). The final sample included 40 apps (20 travel apps and 20 dietary apps).

### App Content

#### Transport Apps

The majority (14/20, 70%) of travel-based apps recorded individual transport behavior with the aim of making users more aware of their travel-related carbon footprint and encouraging more sustainable transport. Additionally, 3 apps (15%) focused on encouraging cycling behavior (Bike da firma, Bikes vs Cars, Cycling 365), 1 app recorded cycling with a fitness/training focus but included personal emissions information (Bike Companion), and 2 apps allowed comparison of different transport modes to make sustainable choices (Green Travel Choice, TripGo). The majority of apps (13/20, 65%) captured multiple transport modes and the associated carbon impact, while 7 apps recorded only 1 mode of transportation such as bicycle, car, bus, or airplane travel (see Multimedia Appendix 1). Although 7 apps reported the calories burned during active transportation, no other health information was included, and no apps directly mentioned the benefit of active transport in reducing sedentary behavior and improving health outcomes. Only 4 apps were completely automated (recorded trip information in the background without having to start and stop trip tracking manually).

#### Diet Apps

Although 1 app focused on the emissions related to the transportation of food (Food Miles Footprint), the majority of dietary apps (19/20, 95%) were simple carbon calculators or surveys that attempted to estimate an individual’s carbon footprint based on inputs from multiple behavioral categories. However, only 8 of these apps (8/19, 42%) displayed the emissions result of food separately from other behaviors (such as household energy use). Dietary inputs typically focused on consumption and purchasing habits as well as information on food transport (eg, local, imported) and farming methods (eg, organic, nonorganic). Meat consumption was the most common dietary habit captured (17/19, 89%), with inputs ranging from simple multiple choice answers such as “no meat,” “some meat,” or “a lot of meat” to the quantity of meat consumed or purchased over a period of time. Only 4 of these apps allowed users to specify the type of meat bought or consumed (Carbon Footprinter, EcoChallenge, MathTappers: Carbon Choices, SustainableI). Fish and dairy consumption were also commonly considered. A total of 8 apps (8/19, 42%) included only a broad representation of dietary behavior with 1 or 2 questions addressing either dietary lifestyle (eg, vegan, vegetarian, meat eater), total food consumption or food transport (eg, local, imported). However, 4 apps captured more detailed information about the consumption of a wide range of food groups such as meat, fish, dairy, rice, bread/cereals/grains, and fruits and vegetables. Only 1 app captured coffee or alcohol consumption; 6 apps captured other information such as food packaging or farming method (eg, organic, nonorganic).

A total of 7 dietary apps (35%) also included information to enhance the user’s knowledge of how dietary habits affect personal GHG emissions, however only 2 apps briefly stated the co-benefits for health and the environment by changing dietary and travel behaviors (Green Plaza and Oroeco).

**Table 3:** App name, developer, type of app, method of data capture, cost, and availability of transport-related apps.

App name	Developer	Type	Data capture	Cost $NZ	Availability
Bikes vs Cars	Fredrik Gertten (WG Film)	Travel + CO_2_^a^	Start/stop	Free	iPhone & Android
Bike Companion	Karlheinz Agsteiner	Travel + CO_2_+ kcal burned^b^	Automatic	Free	Android only
Bike da firma	Bike da firma LTDA-ME	Travel + CO_2_+ kcal burned	Start/stop	Free	iPhone & Android
Changers	Blacksquared GmbH	Travel + CO_2_	Automatic	Free	iPhone & Android
Commute Greener	Pocketweb GmbH	Travel + CO_2_+ kcal burned	Manual	Free	iPhone & Android
Cycling 365	SRM—Societa’ Reti e Mobilita’ Srl	Travel + CO_2_	Start/stop	Free	iPhone & Android
Ecolife	Pipat Apiruktanakorn	Travel + CO_2_+ kcal burned	Start/stop	Free	iPhone & Android
Eco Via	Gregory Carpentier	Travel + CO_2_	Start/stop	Free	Android only
EMission	Kalyanaraman Shankari	Travel + CO_2_+ kcal burned	Automatic	Free	iPhone & Android
Electrip	Volvo Bussar AB	Travel + CO_2_	Start/stop	Free	iPhone & iPad
FuelGood	Energy Saving Trust	Travel + CO_2_	Start/stop	Free	iPhone & Android
Greener Mile	Patrick Hardie	Travel + CO_2_	Start/stop	Free	iPhone only
Green Steps	Alkemy Lab	Travel + CO_2_	Start/stop	Free	iPhone & Android
Green Travel Choice	PocketWeb Ltd	Comparison of travel modes	Manual	$2.59	iPhone & iPad
modalyzer	Innovationszentrum fuer Mobilitaet und gesellschaftlichen Wandel (InnoZ) GmbH	Travel + CO_2_	Automatic	Free	iPhone & Android
My Carbon	International College, KMITL	Travel + CO_2_	Start/stop	Free	Android only
My Open Road	My Open Road Corp	Travel + CO_2_+ kcal burned	Start/stop	Free	iPhone & Android
Singapore G1 Live Green	Balanced Consultancy	Travel + CO_2_+ kcal burned	Start/stop	Free	Android only
TripGo	SkedGo Pty Ltd	Comparison of travel modes	Manual	Free	iPhone & iPad
Vapourz	Oliver Wilson	Travel + CO_2_	Start/stop	Free	iPhone only

^a^Travel + CO_2_ refers to apps that record personal travel and give an estimation of resulting carbon dioxide emissions or savings.

^b^Travel +CO_2_ + kcal burned refers to apps that record personal travel, the associated carbon impact, and kcal burned during active travel.

**Table 4:** App name, developer, type of app, method of data capture, cost, and availability of diet-related apps.

App name	Developer	Type	Data capture	Cost $NZ	Availability
AGE Carbon Calculator	Palacegroup	Carbon Calculator	Multiple choice	Free	Android only
CarbonBuster	Dunman Secondary	Carbon Calculator/educational	Multiple choice	Free	iPhone Only
Carbon Footprinter	Luhui Yan	Carbon Calculator	Numerical input	Free	iPhone Only
CarbonSins	Team Maple Bangalore India	Carbon Calculator	Multiple choice	Free	Android Only
Count Carbon	Don Kershaw	Carbon Calculator	Multiple choice	Free	Android Only
CO_2_ Emission Calculator	FerviDroid	Carbon Calculator	Numerical input	Free	Android only
CO_2_ Footprint	Ship Shape	Carbon Calculator	Multiple choice	Free	iPhone & iPad
EcoChallenge	Raureif GmbH	Carbon Calculator/educational/behavior change	Multiple choice	Free	iPhone Only
eco footprint	Max Gontar	Carbon Calculator	Multiple choice	Free	Android Only
Eco Life Hacks	Anako Dev	Carbon Calculator/educational	Multiple choice	Free	Android Only
ecological footprint	Talents & Treasures, Lda	Carbon Calculator	Multiple choice	Free	Android Only
Food Miles Footprint	BW15 Apps	Calculates emissions due to transportation of food	Numerical/data input	Free	iPhone Only
Green Plaza	Webdunia.com	Carbon Calculator/educational + health	Multiple choice	$1.29	iPhone & iPad
GreenYou	ITAnyplace	Carbon Calculator	Numerical input	iOS–free; Android–$1.29	iPhone & Android
Lotus Greens Carbon Calculator	Lotus Greens Developers Pvt Ltd	Carbon Calculator	Multiple choice/scales	Free	Android Only
Math Tappers: Carbon Choices	HeavyLifters Network Ltd.	Carbon Calculator/educational	Numerical input	Free	iPhone Only
Oroeco	Oroeco Mobile	Carbon Calculator/educational/behavior change + health	Multiple choice/numerical input/scales	Free	iPhone & Android
Residential CO_2_ Alert	Jacques Joosten (Apes apps)	Carbon Calculator	Multiple choice	Free	iPhone Only
SustainableI	DNV GL Business Assurance UK	Carbon Calculator/educational/behavior change	Numerical input	Free	iPhone & Android
VES CO_2_ Tool HD	Veolia Environmental Services	Carbon Calculator/educational	Numerical input/multiple choice	Free	iPhone & iPad

**Figure 2:**
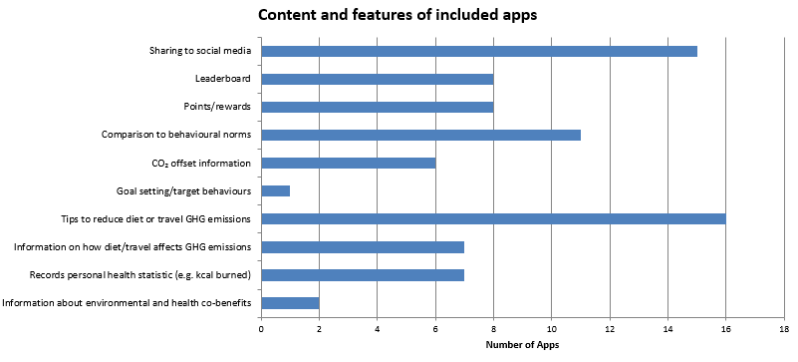
Typical features or information included in travel- or dietary-based apps.

### App Features and Behavioral Change Techniques

Several apps included interactive features to enhance user engagement (Figure 2, Multimedia Appendix 1). Sharing to social media such as Facebook was a feature included in 14 apps (8 transport apps and 6 dietary apps), and 1 dietary app (Carbon Footprinter) allowed sharing to Asian social media such as Sina Weibo. Gamificaton features were incorporated into 9 apps, including reward points and badges for sustainable actions or a leader board where users could compete against others.

Several apps also included features to encourage behavioral change. One app included goal setting, where users set targets for the amount of financial or carbon emissions savings they wanted to make or the calories they wished to burn. A total of 5 travel apps included comparison of different transport modes to help users make more sustainable transport choices, and 16 apps (12 dietary, 4 travel) included specific tips to help users reduce personal GHG emissions. However, the tips included in travel-based apps were typically more implicit and harder to find compared with dietary apps (eg, in the help section of the app). Finally, 11 apps (9 dietary, 2 travel) compared personal emissions with behavioral norms. The majority of these apps (8/11, 73%) used visual comparisons to help user’s easily identify problematic behavior.

### App Quality

In total, 40 apps were rated. One app was rated on both iOS and Android operating systems due to slight differences (My Open Road), and 2 apps were only rated on Android due to issues with functionality on iOS (Green Steps and Eco Life Hacks).

Most apps were rated as acceptable quality (score of 2.50-3.49 out of 5) as determined by the MARS overall mean score; however, 1 travel app was rated as poor quality (score of 1.50-2.49 out of 5), and 7 travel apps and 3 dietary apps were rated as good quality (score of 3.50-4.49 out of 5, see Multimedia Appendix 2). On average, travel apps received higher subjective satisfaction scores compared to dietary apps (*P*=.014, 95% CI 0.13-1.09), and although travel apps tended towards higher overall mean scores (*P=*.08, 95% CI −0.04 to 0.54) and app specific scores (*P*=.15, 95% CI −0.14 to 0.85) compared to dietary apps, they were not significantly different. Travel apps tended to be more engaging (*P*=.03, 95% CI 0.06-0.87) and aesthetically pleasing (*P*=.05, 95% CI −0.003 to 0.88) compared to dietary-based apps, although functionality (*P=*.63, 95% CI −0.51 to 0.31) and information scores (*P=*.15, 95% CI −0.08 to 0.53) were similar between the 2 groups. Free apps were rated slightly higher than paid apps; however, the difference was not significant (*P*=.21, 95% CI −0.21 to 0.92).

Interrater reliability was excellent for the overall mean app quality scores (ICC=0.94, 95% CI 0.87-0.97), the app-specific scores (ICC=0.94, 95% CI 0.87-0.97), and the subjective satisfaction scores (ICC=0.96, 95% CI 0.91-0.98).

## Discussion

### Principal Findings

This study sought to review existing smartphone apps that collect data on individual transport and dietary-related behaviors and estimate personal carbon emissions with a view to determining suitable preexisting tools for assessing changes in behaviors that have health and environment co-benefits. This is the first review to describe features of these types of apps and rate their quality against a valid quality rating tool. Although we found multiple apps for capturing either travel or dietary behavior, we found no single app that fitted inclusion criteria for capturing both behaviors simultaneously, and only 2 apps mentioned the potential co-benefits to health and the environment by changing behaviors. Overall, apps were of mixed quality and few included all the features of being fully automated, including health information, and providing personalized feedback to participants with strategies to make changes to their behavior.

Motorized transport is one of the fastest rising sources of GHG emissions among energy-using sectors and is predicted to rise by 80% between 2007 and 2030 [12,14]. Not only does this pose a grave risk to the environment, it also increases the disease burden associated with sedentary lifestyles [7,12-14]. Strategies to reduce travel-related GHG emissions, such as moving toward more active travel (walking or cycling instead of driving), have demonstrated a reduction in the risk of cardiovascular disease, type 2 diabetes, stroke, dementia, depression, and cancer as a result of improved physical activity levels [7,12,13,16,17]. Moreover, reductions in air pollution associated with alternative transport provide further health benefits by lowering the risk of respiratory disease, cardiovascular disease, and lung cancer [13]. While increased use of active travel may result in small increases in road traffic injuries, it has been repeatedly shown that the potential health benefits heavily outweigh the risks of injury, and risks can be reduced with the implementation of appropriate programs and policies [5,7,13,16].

Even small individual-level changes to travel mode can result in meaningful population-level health benefits [7,13,14,16]. A recent study has estimated that changing just 5% of vehicle travel in Adelaide, South Australia, to cycling results in annual carbon dioxide reductions of 191,313 tons per year, reduces particulate matter and air pollution by 8.5%, and could save 155 deaths and 1991 disability-adjusted life-years associated with chronic disease [13]. However, the largest benefit to both public health and the environment results from the combination of significant increases in active travel and reduced reliance on motorized travel [12,13,17]. Changing 40% of vehicle travel to alternative modes, such as public transportation and cycling, is estimated to potentially reduce the total disease burden attributed to physical inactivity by 55% [13]. Despite the abundance of evidence showing the potential health benefits of small changes to travel mode, none of the reviewed transport apps provided adequate information regarding the impact of personal travel behavior on health and the risk of NCDs (other than the number of calories burned during active travel).

Although the environmental effects of motorized transport may be widely acknowledged, individuals may be less aware of the indirect effects of individual dietary consumption on climate change. Agricultural activities and deforestation contribute more to global GHG emissions than transport, thereby exacerbating climate change and its subsequent effects on health, including food yields [6,19,21,22]. Despite the significant impact that individual dietary behavior may have on climate change, we found no apps that were solely focused on estimating personal emissions attributed to dietary behavior. Diet-related apps included in this review were mostly carbon calculators that addressed multiple behavioral categories. Although some apps attempted to measure the environmental impact of dietary behavior in more depth, few apps adequately captured all aspects contributing to personal diet-related emissions. Kim and Neff [34] reported similar findings with only 25% of all carbon calculators including a diet component, of which most addressed only 1 diet-related behavior. The limited scope of dietary behaviors captured by apps included in this review may be attributed to the trade-off between the accuracy of measurement and the burden of significant manual data input.

Livestock production alone accounts for 18% of global GHG emissions, and global meat and dairy consumption (and therefore livestock production) are predicted to double from 229 million tons in 1999-2001 to 465 million tons in 2050 [21]. Therefore, meat consumption was appropriately one of the primary considerations of most diet-related apps in this review, with some also considering milk or dairy consumption. Ruminant animals (eg, cattle, sheep, and goats) account for the majority of livestock’s GHG emissions due to significant methane emissions from enteric fermentation compared to monogastric animals (eg, pigs and poultry) [25]. However, only 4 of the included dietary apps allowed users to specify the type of meat consumed. Rice consumption was considered by 4 of the included apps, as its cultivation also contributes to methane emissions, which are more damaging to the environment than carbon emissions [19]. Additionally, since the majority of emissions “beyond the farm gate” come from the transportation of food [19], some apps addressed the food source, namely whether it was imported or grown locally.

Changes to dietary behavior such as reducing the intake of meat and dairy products are necessary to achieve meaningful reductions in food-related GHG emissions [6,19,21]. However, these changes also have potential health benefits, especially within high-income countries where dietary excess and the increase in availability of animal-based foods high in saturated fat contribute to the burden of NCDs [6,8,9,19,21]. Furthermore, if GHG emissions from livestock production could be curtailed, a reduction in red meat consumption in high-income countries could in the short term allow for slight increases in very low-income countries, addressing issues of mal- and undernutrition, thereby providing health benefits for all [6,21]. However, in the face of large projected increases in meat consumption within the developing world, not only will changes be necessary in high-income countries, but low and middle-income countries will have to moderate their intakes per capita [19]. Only 2 of the dietary apps included in this review made reference to the potential health benefits of dietary changes. The Oroeco app provided an explanation of why a reduction in the consumption of animal products is not only good for the environment but also reduces the risk of obesity and associated NCDs (heart disease, diabetes, and many cancers), while the Green Plaza app only mentioned that these changes are good for your health, your budget, and the planet.

Only 10 out of 40 apps (25%) included in this review explicitly encouraged behavior change by rewarding sustainable behavior or providing clear instruction to change behavior (eg, try walking, cycling, or using public transport instead of driving). The remaining apps were more implicit, focusing on creating awareness of the impact of current behavior with some providing information, tips or sustainable alternatives with potential to encourage behavior change. Of the reviewed apps, dietary apps typically included more tips to help users reduce emissions and were more likely to provide clarity regarding behavioral norms compared with travel apps. However, although a few apps provided many good quality tips or recommendations to mitigate personal emissions (Oroeco, VES CO2 Tool, Eco Life Hacks, SustainableI, Carbon Buster), most apps provided very few or no tips with no explanation or background information, potentially reducing their effectiveness in changing users’ attitudes and ultimately behavior. Apps that rated highest typically included features to maximize user interaction and engagement such as sharing to social media (Facebook) and elements of gamification (leader board, competition/comparison to other users, or rewards) in addition to producing reasonable estimates of personal emissions, providing good quality information and strategies to mitigate personal GHG emissions. Technology employing behavior change techniques such as self-monitoring, feedback, comparison with behavioral norms, peer influence/social networking, or gamification has been promising in evoking changes in attitudes and behavior in both travel and health contexts [35-38]. Future research is needed to determine which features or techniques within smartphone apps are required to trigger and sustain behavior change, resulting in climate change and health co-benefits.

Previous research has identified that carbon calculators tend to be meaningless unless represented in a more tangible form such as comparison to social norms/target behaviors or showing the direct environmental impact of individual actions [39]. Although the majority of apps included in this review provided emissions results in tons, kilograms, or carbon dioxide equivalents annually, which may be difficult for users to interpret, several apps used more effective communication methods. The Changers and VES CO2 Tool HD apps incorporated real-world representations of carbon dioxide emissions or savings (eg, “this is equivalent to one cycle of laundry at 60°C or watching TV for a year”), 8 apps showed clear comparison to behavioral norms (ie, clear visual representation), 2 apps represented emissions as the number of planets required to sustain behavior if all humans had the same impact, and 5 apps represented emissions in terms of the number of trees required to achieve carbon neutrality.

### Strengths and Limitations

A strength of this review is that, to our knowledge, it is the first study of its kind to synthesize available smartphone apps for capturing personal travel and dietary behavior with an emphasis on GHG emissions and NCDs. It also used a validated quality rating tool and described the presence or absence of behavior change techniques required to initiate and maintain travel and dietary behavior. This study, however, was limited to apps for Android or iOS devices available at the time of the search and therefore does not include apps for Windows phones or other technologies. Furthermore, new apps are constantly being developed and existing apps improved, thus our review is relevant to the versions available at this time.

### Future Recommendations

Future apps should incorporate information and features to promote the health benefits of strategies to reduce personal GHG emissions. This could potentially result in greater behavioral change by appealing to the user’s self-interest in achieving optimal personal health in addition to enabling contributions to wider global challenges such as climate change [39]. Potential exists to include health monitoring data (eg, heart rate, arterial oxygen saturation) by linking with other devices, thereby guiding users towards improved health and wellbeing while simultaneously reducing personal GHG emissions. More information could be incorporated to ensure at-risk populations are still meeting dietary guidelines despite changes in diet. For example, recommendations to reduce meat consumption could be paired with information about alternative protein and iron sources, possibly including recipes, especially for menstruating women, high intensity athletes, or anyone cooking for growing children. Apps should also simultaneously and automatically track all forms of physical activity and travel, giving a clear indication of health gain from active travel.

In addition, potential exists to include a broader scope of dietary and transport behaviors to more accurately and dynamically capture personal carbon emissions. Diet-based apps should ideally capture all aspects of food-related emissions from farm to waste, for all foods consumed (especially high-impact foods such as animal products and rice), and specify the type of meat consumed (eg, red meat from ruminants vs poultry or pork). Travel apps have potential for greater integration with multimodal transportation methods such as plane, rail, bus, car, motorcycle, and boat/ferry travel.

Studies suggest that participants value accuracy in tracked data [40] but view manual data entry as burdensome and may forget to record smaller travel trips if required to do so [27]. Therefore future apps should capture data continuously and ubiquitously, with minimal manual participant input. However, thought should be given to the trade-off between data accuracy, using continuous automatic GPS tracking, and the preservation of limited battery energy. App users are very aware of battery power consumption and may delete apps with high battery use, deeming them unnecessary [28,40]. Therefore, the inconvenience of frequent battery recharge may outweigh any benefit of using auto-geolocation. Furthermore, care should be taken to protect the user’s privacy by granting control of the tracking feature such as enabling the user to set “sensitive areas” or “sensitive times” [28].

The ability for apps to deliver personalized advice or information “on the go” has been identified as a valued feature among app users [40]. However, personal carbon calculators may produce abstract scores, making them difficult to interpret and understand. Furthermore, the inconsistencies in carbon calculator scores and unclear methodology makes them difficult to compare, standardize, and benchmark [34,41]. Future apps should consider the use of more effective ways to communicate personal emissions data in a way that users will understand. Future work should also include improved visibility and transparency of data sources and development of new metrics and computation methodology that provide insightful and valuable information on people’s personal carbon emissions and potential health co-benefits associated with dietary and physical activity-related behaviors.

Finally, future apps could include more techniques and interactive features (such as sharing to social media and app communities) to maximize participant engagement, promote sustained use, and ultimately evoke behavior change.

### Conclusion

This review revealed multiple apps for capturing either dietary or travel behavior and estimating the associated carbon cost. However, we found no single app that adequately captured both behaviors simultaneously and addressed the potential co-benefits for the environment and health by changing these behaviors. Overall, existing apps are of mixed quality, and none included all of the features of being fully automated, providing adequate health information and personalised feedback to participants with strategies to make changes to their behavior.

The ubiquity of smartphone use, their inbuilt sensors (accelerometers, GPS), and their computational power makes them an ideal device for collecting personal data on travel and dietary behaviors as well as personal carbon emissions and using these data to provide “just-in-time” interventions to evoke behavior change. Future research is needed to determine the potential of such approaches to change behavior.

## Appendix

Multimedia Appendix 1 Content and features of all included apps.

Multimedia Appendix 2 Mobile Application Rating Scale (MARS) quality scores of the 2 evaluators for all included apps.

## Abbreviations

CO2: carbon dioxide
GHG emissions: greenhouse gas emissions
GPS: Global Positioning System
ICC: intraclass correlation coefficient
MARS: Mobile Application Rating Scale
NCD: noncommunicable disease
