# Supplementary material for: Smartphone Apps for Measuring Human Health and Climate Change Co-Benefits: A Comparison and Quality Rating of Available Apps
Source: JMIR Mhealth Uhealth. 2016 Dec 19;4(4):e135. doi: 10.2196/mhealth.5931 (PMC5206483; doi:10.2196/mhealth.5931)
Supplement: Multimedia Appendix 2 [file mhealth_v4i4e135_app2.pdf]

Table 1 - MARS Quality Scores for Transport Apps

App Title	iOS or Android	Engagement		Functionality		Aesthetics		Information		Subjective score		App Specific Score		Overall Mean Score	
		Rater 1	Rater 2	Rater 1	Rater 2	Rater 1	Rater 2	Rater 1	Rater 2	Rater 1	Rater 2	Rater 1	Rater 2	Rater 1	Rater 2
**Bikes vs Cars**	iOS	3.00	2.20	5.00	4.80	3.67	4.70	3.00	2.40	3.00	2.80	2.40	2.00	3.67	3.50
**Bike Companion**	Android	2.00	-	2.25	-	2.33	-	2.20	-	2.50	-	1.20	-	2.20	
**Bike da firma**	iOS	3.40	-	3.75	-	4.00	-	3.00	-	3.75	-	2.60	-	3.54	-
**Changers**	iOS	3.80	3.60	4.00	4.50	4.33	4.30	3.80	3.40	3.75	3.50	3.60	3.20	3.98	3.90
**Commute Greener**	iOS	2.00	-	2.75	-	2.33	-	3.00	-	1.75	-	2.80	-	2.52	-
**Cycling 365**	iOS	2.00	2.00	3.25	3.30	3.00	2.00	3.17	3.20	2.25	3.50	1.60	1.20	2.86	2.60
**Ecolife**	iOS	3.00	2.60	4.50	4.30	4.00	4.30	3.00	3.50	4.00	4.00	3.60	3.40	3.63	3.70
**Eco Via**	Android	2.60	2.60	4.00	4.30	3.67	3.70	3.17	3.50	2.75	3.50	2.80	2.00	3.36	3.50
**E-Mission**	iOS	2.00	-	3.00	-	2.67	-	2.60	-	1.75	-	1.40	-	2.57	-
**Electrip**	iOS	2.60	2.20	4.75	4.50	4.00	4.00	2.80	2.60	2.25	2.30	2.20	1.40	3.54	3.30
**Fuel Good**	iOS	3.00	2.40	4.75	4.80	3.33	3.30	3.83	3.30	3.50	3.50	3.60	3.40	3.73	3.50
**Greener Mile**	iOS	3.00	3.20	4.50	4.50	3.00	3.30	3.50	4.00	3.75	4.00	3.20	2.80	3.50	3.80
**Green Steps**	Android	2.00	1.80	4.25	4.30	3.33	3.00	2.60	2.50	1.75	1.50	1.60	1.00	3.05	2.90
**Green Travel Choice**	iOS	2.20	2.20	4.25	4.30	2.67	2.30	3.20	2.60	2.50	2.80	2.80	2.60	3.08	2.90
**modalyzer**	iOS	2.80	-	4.00	-	3.33	-	3.00	-	2.25	-	2.40	-	3.28	-
**My Carbon**	Android	2.40	-	3.75	-	3.33	-	3.00	-	2.00	-	2.00	-	3.12	-
**My Open Road**	Android	3.80	3.40	3.50	3.30	3.67	3.30	3.20	3.50	3.25	3.80	3.20	2.60	3.54	3.40
**My Open Road**	iOS	3.60	3.40	3.75	3.80	4.00	2.70	3.17	3.40	3.75	4.00	3.20	2.80	3.63	3.20
**Singapore G1 Live Green**	Android	2.20	-	3.50	-	3.00	-	2.40	-	1.75	-	2.00	-	2.78	-
**TripGo**	iOS	3.20	3.40	4.25	3.80	3.33	3.70	3.25	3.30	3.50	4.30	2.20	2.20	3.51	3.60
**Vapourz**	iOS	2.40	2.20	4.75	5.00	3.33	3.30	3.20	2.80	2.50	2.30	1.80	1.40	3.42	3.30

*Shaded green = good quality, no shading = acceptable quality, shaded grey = poor quality

Table 2 - MARS Quality Scores for Diet Apps

App Title	iOS or	Engagement	Functionality	Aesthetics	Information	Subjective	App Specific	Overall Mean

	Android									score		Score		Score	
		Rater 1	Rater 2	Rater 1	Rater 2	Rater 1	Rater 2	Rater 1	Rater 2	Rater 1	Rater 2	Rater 1	Rater 2	Rater 1	Rater 2
**AGE Carbon Calculator**	Android	1.60	-	3.50	-	2.67	-	2.67	-	1.75	-	1.40	-	2.61	-
**Carbon Buster**	iOS	2.00	2.00	4.50	4.30	3.00	3.30	2.80	2.60	2.50	2.50	2.60	3.00	3.07	3.10
**Carbon Footprinter**	iOS	1.80	1.60	4.00	4.80	2.33	2.00	3.00	2.70	2.00	2.00	2.00	1.60	2.78	2.80
**CarbonSins**	Android	1.18	-	2.75	-	2.00	-	2.00	-	1.50	-	1.00	-	1.98	-
**Count Carbon**	Android	1.60	1.40	5.00	5.00	1.67	2.00	1.80	1.80	1.50	1.50	1.00	1.00	2.52	2.60
**CO_2 Emission Calculator**	Android	1.40	-	5.00	-	1.30	-	2.50	-	1.50	-	1.20	-	2.55	-
**CO_2 Footprint**	iOS	2.20	2.20	5.00	5.00	3.00	3.70	2.20	3.20	1.50	2.50	1.40	2.00	3.10	3.50
**EcoChallenge**	iOS	3.40	-	4.00	-	3.67	-	3.33	-	3.00	-	3.00	-	3.60	-
**eco footprint**	Android	2.20	-	3.75	-	2.00	-	2.75	-	2.25	-	1.40	-	2.68	-
**ECO LIFE HACKS**	Android	2.00	-	3.75	-	3.00	-	2.80	-	2.00	-	2.00	-	2.89	-
**ecological footprint**	Android	1.40	-	4.25	-	3.00	-	2.80	-	1.75	-	1.00	-	2.86	-
**Food Miles Footprint**	Android	2.80	2.20	3.00	3.80	3.33	4.00	3.20	2.40	2.25	2.50	2.20	2.00	3.08	3.10
**Green Plaza**	iOS	2.20	2.00	4.25	3.80	2.67	2.70	2.40	2.30	2.00	1.80	1.80	2.00	2.88	2.70
**GreenYou**	iOS	1.80	1.60	4.00	3.80	2.33	2.00	2.67	2.30	1.75	1.50	1.60	1.00	2.70	2.40
**Lotus Greens Carbon Calculator**	Android	2.60	1.80	3.75	3.50	3.00	3.00	3.00	3.00	2.25	2.00	1.60	1.20	3.09	2.80
**Math Tappers: Carbon Choices**	iOS	2.40	1.80	3.25	4.30	3.00	2.70	3.83	3.00	3.00	2.80	3.40	3.00	3.12	3.00
**Oroeco**	iOS	3.60	3.20	3.75	4.00	4.00	4.70	4.00	3.70	3.75	3.80	3.60	3.80	3.84	3.90
**Residential CO_2 Alert**	iOS	2.80	2.30	3.50	4.80	3.33	3.70	2.20	2.00	2.00	2.30	1.80	2.00	2.96	3.20
**SustainableI**	iOS	3.00	-	3.50	-	3.33	-	3.60	-	2.50	-	2.80	-	3.36	-
**VES CO_2 Tool HD**	iOS	3.60	3.40	4.50	4.50	3.67	4.00	4.00	3.40	3.75	3.80	3.40	3.00	3.94	3.80

*Shaded = good quality, no shading = acceptable quality
